# Changes in Rat Brain Tissue Microstructure and Stiffness during the Development of Experimental Obstructive Hydrocephalus

**DOI:** 10.1371/journal.pone.0148652

**Published:** 2016-02-05

**Authors:** Lauriane Jugé, Alice C. Pong, Andre Bongers, Ralph Sinkus, Lynne E. Bilston, Shaokoon Cheng

**Affiliations:** 1 Neuroscience Research Australia, Margarete Ainsworth Building, Randwick, Australia; 2 University of New South Wales, School of Medical Sciences, Wallace Wurth Building, Kensington, Australia; 3 University of New South Wales, Biological Resources Imaging Laboratory, Lowy Cancer Research Centre, Kensington, Australia; 4 King’s College London, Chair in Biomedical Engineering, Imaging Sciences & Biomedical Engineering Division Kings College, St. Thomas’ Hospital, London, United Kingdom; 5 University of New South Wales, Prince of Wales Clinical School, Edmund Blacket Building, Kensington, Australia; 6 Macquarie University, Department of Engineering, Faculty of Science, Macquarie University, Sydney, Australia; Brighton and Sussex Medical School, UNITED KINGDOM

## Abstract

Understanding neural injury in hydrocephalus and how the brain changes during the course of the disease in-vivo remain unclear. This study describes brain deformation, microstructural and mechanical properties changes during obstructive hydrocephalus development in a rat model using multimodal magnetic resonance (MR) imaging. Hydrocephalus was induced in eight Sprague-Dawley rats (4 weeks old) by injecting a kaolin suspension into the cisterna magna. Six sham-injected rats were used as controls. MR imaging (9.4T, Bruker) was performed 1 day before, and at 3, 7 and 16 days post injection. T2-weighted MR images were collected to quantify brain deformation. MR elastography was used to measure brain stiffness, and diffusion tensor imaging (DTI) was conducted to observe brain tissue microstructure. Results showed that the enlargement of the ventricular system was associated with a decrease in the cortical gray matter thickness and caudate-putamen cross-sectional area (*P* < 0.001, for both), an alteration of the corpus callosum and periventricular white matter microstructure (CC+PVWM) and rearrangement of the cortical gray matter microstructure (*P* < 0.001, for both), while compression without gross microstructural alteration was evident in the caudate-putamen and ventral internal capsule (*P* < 0.001, for both). During hydrocephalus development, increased space between the white matter tracts was observed in the CC+PVWM (*P* < 0.001), while a decrease in space was observed for the ventral internal capsule (*P* < 0.001). For the cortical gray matter, an increase in extracellular tissue water was significantly associated with a decrease in tissue stiffness (*P* = 0.001). To conclude, this study characterizes the temporal changes in tissue microstructure, water content and stiffness in different brain regions and their association with ventricular enlargement. In summary, whilst diffusion changes were larger and statistically significant for majority of the brain regions studied, the changes in mechanical properties were modest. Moreover, the effect of ventricular enlargement is not limited to the CC+PVWM and ventral internal capsule, the extent of microstructural changes vary between brain regions, and there is regional and temporal variation in brain tissue stiffness during hydrocephalus development.

## Introduction

Hydrocephalus is a common structural neurological disorder [1, 2]. The disease is characterized by enlargement of cerebral ventricles, caused by the abnormal build-up of cerebrospinal fluid (CSF), which compresses the surrounding tissue and results in functional deficits and cognitive impairments [3–5]. Previous studies have shown that damaged brain tissue could be related to symptoms in hydrocephalic patients [6–10], but the detailed mechanisms of neural injury are poorly understood. How neural injury progresses during the course of the disease is not known, even though this is essential to track disease progression and improve clinical management [11].

To understand changes in the brain parenchyma and elucidate mechanisms of neural injury, it is important to understand how the brain deforms during the development of hydrocephalus and how this affects the tissue microstructure and mechanical properties. It is widely assumed that brain mechanical properties play an important role during the development of the disease and that they change during ventricular enlargement [12]. However, it is unknown whether mechanical properties change due to tissue deformation because compression increases the stiffness of neural tissue [13] or if underlying differences in brain stiffness promote ventricular enlargement, perhaps due to higher brain compliance [14, 15]. For example, in normal pressure hydrocephalus, brain stiffness has been reported to be lower than in healthy controls [16, 17]. However, how brain tissue mechanical properties change in obstructive hydrocephalus is unclear. They have been estimated from measurements of the intracranial system's compensatory reserve [18] and indentation techniques [19] in animal models. Although both techniques are far from ideal, as they either include the stiffness of other intracranial structures or are limited only to the superficial regions, both suggested that brain tissue stiffness is increased in the presence of enlarged ventricles.

This study aims to understand how tissue microstructure and mechanical properties change during ventricular enlargement in obstructive hydrocephalus using multimodal magnetic resonance (MR) imaging. While standard anatomical MR imaging can depict ventricular size [20], other methods can give complementary information. First, diffusion tensor imaging (DTI) gives valuable insight into brain tissue microstructure [21, 22]. This imaging technique can delineate axonal organization of the white matter based on the anisotropic diffusion of water and can also provide structural information in the gray matter regions which are less anisotropic [23–25]. Second, MR elastography is an emerging technique which measures mechanical properties from the velocity and attenuation of shear wave propagation using a motion-sensitive MR imaging sequence [26]. MR elastography has been applied to the human [27–30] and rodent brain [31–35].

This study uses an animal model, reminiscent of obstructive hydrocephalus post-meningitis, in which hydrocephalus induction is well controlled [36–38]. We describe brain deformation, microstructural and mechanical properties changes occurring during the early stages of disease development using anatomical MR imaging, DTI and MR elastography respectively. We used an animal model because it is difficult to investigate early changes in human patients, as they are not scanned until symptoms appear, and this usually occurs only after significant enlargement of the cerebral ventricles. We hypothesize that (1) the impact of ventricular enlargement on the parenchyma is not limited to the corpus callosum and periventricular white matter (CC+PVWM) in the early stages of the disease development, (2) there is a regional variation with time for the microstructural changes, and (3) brain mechanical properties are affected by the development of obstructive inflammatory hydrocephalus.

## Materials and Methods

Young rats injected with a kaolin-suspension into the cisterna magna have been widely used as a model of obstructive inflammatory hydrocephalus [39–41]. This method involves the obstruction of CSF pathway by causing inflammation of the arachnoid mater. The experimental protocol was approved by the local animal care and ethics committee (University of New South Wales, Kensington, Australia, number 12/21B).

### Experimental Procedures

Fourteen Sprague-Dawley rats (female, 3 weeks old, Animal Resources Center, Canning Vale, WA, Australia) were acclimatized for one week before the beginning of the protocol. They were housed in cages on a 12 hour light/dark cycle and had free access to water and pellet food. Rats were anaesthetized with 1.5% isoflurane in 100% oxygen delivered at a constant rate of 1L/min via a face mask. Under aseptic conditions, the tip of the 27 gauge needle was inserted into the cisterna magna and either thirty microliters of suspension of kaolin (Sigma-Aldrich, Castle Hill, Australia) 25% w/v in 0.9% saline at the rate of 6 μL/s was injected (hydrocephalic group, n = 8) or no suspension was injected (controls n = 6) [37]. After surgical recovery, daily subcutaneous injections of analgesic (Temgesic^®^, buprenorphine hydrochloride, 0.05 mg/kg, Cenvet, Blacktown, Australia) were given 2–3 times as needed for pain.

### Image acquisition, data reconstruction and analysis

Rats were imaged using a 9.4T Bruker (Ettlingen, Germany) BioSpec Avance III 94/20 magnetic resonance microimaging system equipped with BGA-12S HP gradients with maximum strength 660 mT/m and slew rate 4570 mT/s (Biological Resources Imaging Laboratory, University of New South Wales, Australia). Signal was generated and received using an 86 mm ID quadrature volume transmitter in combination with a 20 mm diameter single loop surface coil (Bruker) that was placed on top of the rat skull.

Animals were imaged at 4 time points: Baseline (one day pre injection, day-1), three, seven, and sixteen days post kaolin / sham injection. The rats were placed in prone position, head first in the scanner, and anaesthetized with 1%-1.5% isoflurane (Pharmachem, Eagle Farm, Australia) in 100% oxygen delivered at a constant rate of 1L/min via a face mask. Body temperature and breathing rate were monitored in real time using an animal life monitoring system (SA Instruments, Inc., Stony Brook, NY, USA). Body temperature was maintained between 36°C-37°C using a heated blanket.

**Fig 1:**
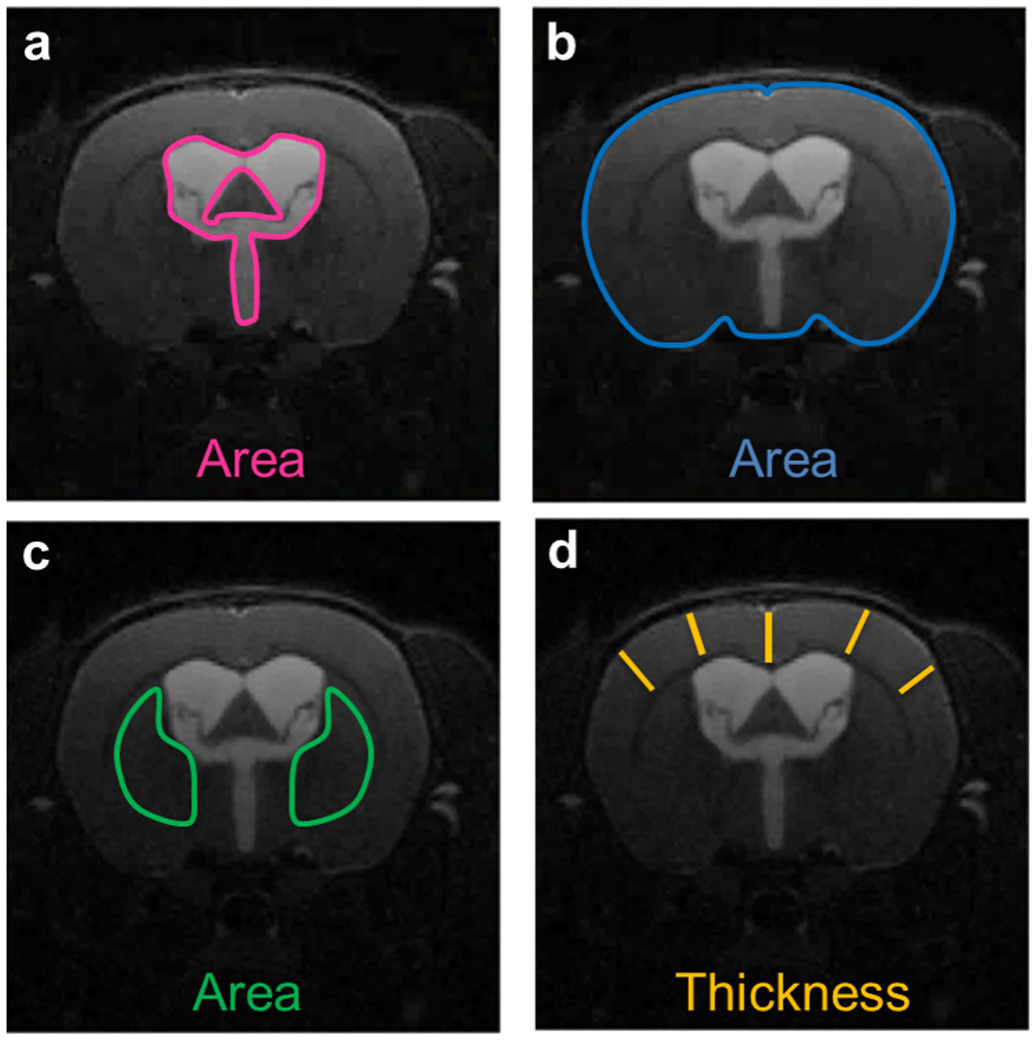
Brain deformation variables. Brain deformation was assessed during hydrocephalus development by quantifying changes in cross-sectional areas of the ventricular system (pink ROI, a), the whole brain (blue ROI, b), the caudate-putamen as the sum of the right and left caudate-putamen (green ROIs, c), and the thickness of the cortical gray matter as measured from the pial surface to the subcortical white matter (averaged over 5 locations, d) from the MR anatomical images.

**Fig 2:**
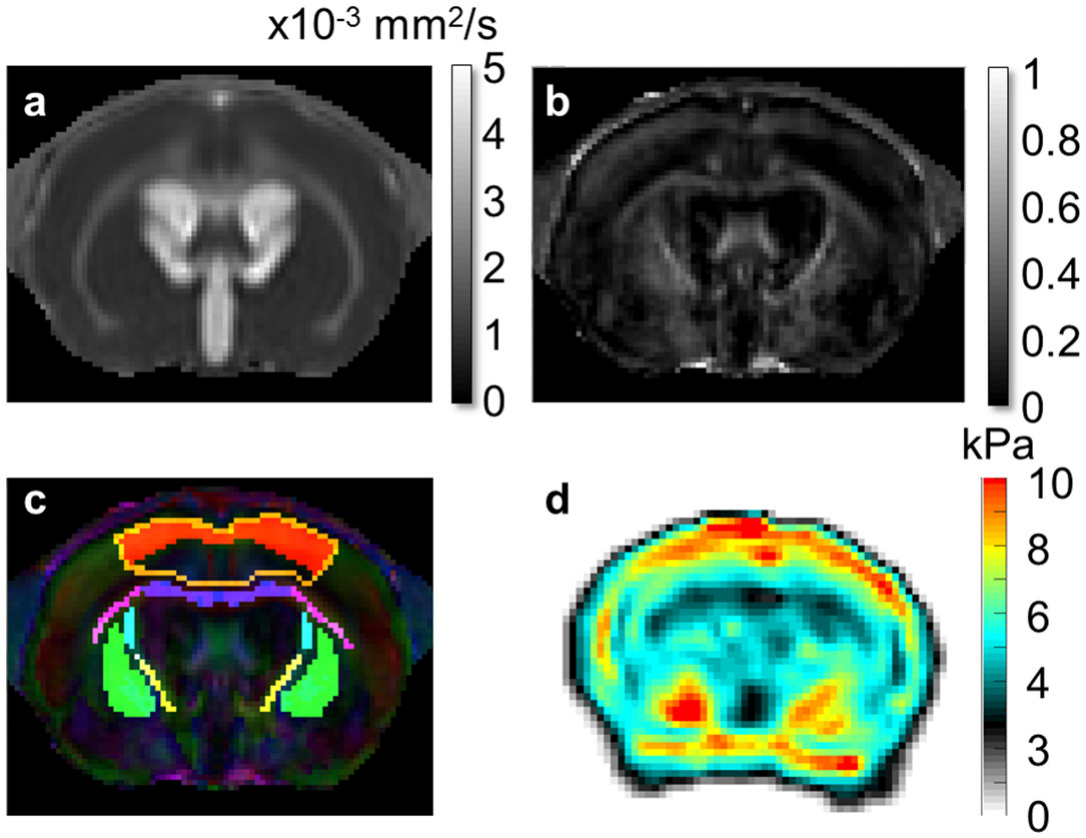
Typical diffusion and shear modulus maps of an hydrocephalic rat brain obtained with a 9.4 T (Bruker). (a) Mean diffusivity map (× 10^−3^ mm^2^/s). (b) Fractional anisotropy map. (c) Colored fractional anisotropy with seven ROIs: 1- corpus callosum and periventricular white matter (CC+PVWM, i.e area of the corpus callosum extending all the way to the dorsolateral angle of the lateral ventricle, violet), 2- external and 3- ventral internal capsule (pink and yellow respectively), 4- cortical gray matter (i.e area over the corpus callosum and the roof the lateral ventricles from the periventricular white matter to the pial surface, golden), 5- upper cortical gray matter (i.e top layers of the cortical gray matter excluding edematous region, red), 6- caudate-putamen (i.e average over left and right caudate-putamen, green), 7- dorsal internal capsule (turquoise). (d) Shear modulus (G*) map at 800 Hz (kPa).

The imaging protocol consisted of:

1. Localizer scan: two-dimensional anatomical images were acquired using a rapid acquisition with relaxation enhancement sequence to localize the foramina of Monro with the following parameters: repetition time / echo time = 3000 ms /9.5 ms, rapid acquisition with relaxation enhancement factor = 16, twelve signal averages, one repetition, 9 contiguous sections, field of view = 19.2 mm × 19.2 mm, slice thickness = 600 μm, in-plane resolution = 300 μm × 300 μm, acquisition time = 2 minutes 24 seconds.
2. Anatomical T2-weighted axial images of the ventricular system, cortical gray matter and caudate-putamen were obtained using a two dimensional rapid acquisition with relaxation enhancement sequence (TurboRARE), with the following parameters: repetition time / echo time = 3000 ms/9.5 ms, rapid acquisition with relaxation enhancement factor 16, twenty four signal averages, one repetition, nine contiguous slices, field of view = 19.2 mm × 19.2 mm, slice thickness = 300 μm, in-plane resolution = 150 μm × 150 μm, acquisition time = 9 minutes 36 seconds.
  T2-weighted images were used to assess brain deformation by measuring over the middle slice acquired centered on the foramina of Monro: (1) cross-sectional areas of the ventricular system, whole brain, caudate-putamen, and (2) thickness of the cortical gray matter using ImageJ (version 1.47, National Institutes of Health, USA) (Fig 1).
3. DTI data were obtained using a four-shot echo planar spin echo sequence triggered on the inspiration peak from the respiration system to minimize respiration-induced motion artefacts. The following parameters were used: repetition time / echo time = 3800 ms /23.5 ms, two signal averages, one repetition, thirty gradient directions with a *b* factor = 670 s/mm^2^ and five b_0_-reference scans (without diffusion gradient switching), thirteen slices with slice thickness 700 μm, 300 μm slice gap, field of view = 25 mm × 25 mm,in-plane resolution = 195 μm × 195 μm, acquisition time = 17 minutes 45 seconds.
  Mean diffusivity (MD) and fractional anisotropy (FA) maps were calculated on a pixel-wise basis using DSI Studio (September 2014 version, Department of Biomedical Engineering, Carnegie Mellon University, PA, USA) by calculating eigenvectors of the diffusion matrix [21, 22] (Fig 2). Any images with motion artefacts were excluded. Average mean diffusivity and fractional anisotropy were obtained over the middle slice acquired centered on the foramina of Monro from seven different regions of interest (ROIs) drawn on the colored fractional anisotropy map and they are: (1) corpus callosum and periventricular white matter (CC+PVWM), (2) external capsule (ec), (3) ventral internal capsule (VIC), (4) cortical gray matter (CGM), (5) upper cortical gray matter, (6) caudate-putamen (CP), and (7) dorsal internal capsule (Fig 2).
4. MR elastography data was obtained using a modified two dimensional multi-slice spin echo sequence with sinusoidal motion sensitizing gradients (570 mT/m) synchronized with continuous sinusoidal mechanical vibration at 800 Hz [26, 28, 42]. Mechanical vibrations were transmitted to the brain via a carbon fiber rod which was connected to an electromagnetic shaker (Brüel & Kjaer, Nærum, Denmark) located outside the MR scanner [35, 43, 44]. The rod was connected to a custom-made tooth bar that fits tightly to the rat’s incisors to ensure that mechanical wave can propagate effectively throughout the brain via the teeth and skull. By consecutively applying the gradients to three axis, the three dimensional propagation of the mechanical wave inside the brain was phase encoded over eight time-points distributed equally over one vibration period. The MR elastography data were collected in matched geometry with high resolution T2-weighted images with repetition time / echo time 2813 ms /29 ms, one signal average, field of view = 19.2 mm × 19.2 mm, slice thickness = 300 μm, in-plane resolution = 300 μm × 300 μm, acquisition time for each of the three directions 24 minutes. Data were considered reliable if the local total wave amplitude was greater than 0.2 μm. This value corresponded to three times the total amplitude of the noise level (i.e. 0.06 μm) based on pilot acquisitions where no mechanical wave was applied.
  The magnitude of the complex shear modulus (hereafter called ‘shear modulus’, G*) represents the tissue stiffness and was calculated at each voxel by numerically solving the wave equation for acoustic propagation through a linear viscoelastic medium [45], using custom software [28, 46] (Fig 2). Shear moduli were averaged over the middle three of the nine acquired slices. The mean shear modulus was calculated over the same regions used to measure the diffusion properties for the cortical gray matter (ROI 4) and caudate-putamen (ROI 6). ROIs were drawn on the T2 weighted anatomical images and then transferred to the shear modulus map.

Intracranial pressure, blood pressure and arterial blood gases of rats were not measured in this study, due to difficulties in combining these procedures with the MR elastography setup in the small bore MRI system used for this study. Additional invasive monitoring also would have imposed more stress on the animals, and further increased the risks of complications.

### Histology

After the final scan, rats were placed under deep anaesthesia (2.5%-3% of isoflurane (Pharmachem, Eagle Farm, Australia) in 100% oxygen at the rate of 1L/min) and euthanized by intracardiac perfusion of 100 mL of phosphate buffered saline 1X (Sigma-Aldrich, Castle Hill, Australia) and then 100 mL of 10% buffered formalin solution (Thermo Fisher Scientific Australia Pty Ltd, Scoresby Vic, Australia). The brains were fixed for 24 hours in 10% buffered formalin solution and stored at room temperature in Ethanol (70% v/v, Sigma-Aldrich, Castle Hill, Australia).

The brains were embedded in paraffin and axial sections of five μm thickness were cut. The slides were stained using Luxol Fast Blue with Cresyl Violet on the Leica Auto—stainer XL (Leica Biosystems, Wetzlar, Germany) to visualize neuronal organization of the brain tissue and myelination in the white matter. High resolution images (40 × magnification) were obtained by scanning the glass microscopic slides using an Aperio Scan Scope XT Slide Scanner (Leica Biosystems, Wetzlar, Germany).

### Statistical analyses

Mean and standard deviation for each variable were calculated at each time point. Longitudinal changes in (1) brain deformation (i.e cross-sectional area of the ventricular system, whole brain, and caudate-putamen, and the thickness of the cortical gray matter); and (2) indicators of brain tissue microstructure (mean diffusivity and fractional anisotropy) were examined using a repeated measures two-way ANOVA analysis to test the interaction between the two groups of rats (control and hydrocephalic rats). Post-hoc Tukey’s test was used to compare variables between time points in each group of rats, and Sidak’s test to compare the two groups of rats at each time point. The effect of hydrocephalus development on the brain mechanical properties was assessed using a multiple t-test approach (α = 0.05, unpaired) to compare the two groups of rats at each time-point because 10 of 54 total MR elastography measurements were excluded due to poor quality (wave amplitude inferior to 0.2 μm). Statistical tests were performed with GraphPad Prism (version 6.01, San Diego, California, USA).

Finally, generalized estimating equations (GEE) were used to assess linear relationships between variables while accounting for repeated measured design, using SPSS (v22, IBM, Armonk, New York, USA).

## Results

Hydrocephalic rats lost an average of 12 ± 5% of their bodyweight after surgery but recovered from weight loss over time while the control rats gained weight gradually over time. One hydrocephalic rat was euthanized three days post-kaolin injection due to post-operative pain. Data obtained from this rat at baseline and day three are included in the box plot of the graphs, and the data tables reported in supporting information (S1–S4 Tables) and considered in the statistical analysis except the repeated measures two-way ANOVA tests.

At baseline, no statistically significant differences were observed between control and hydrocephalic rats for brain deformation, mean diffusivity, fractional anisotropy (see below) or mechanical properties (t-test; shear modulus: cortical gray matter *P* = 0.94, caudate-putamen *P* = 0.46).

### Brain deformation

**Fig 3:**
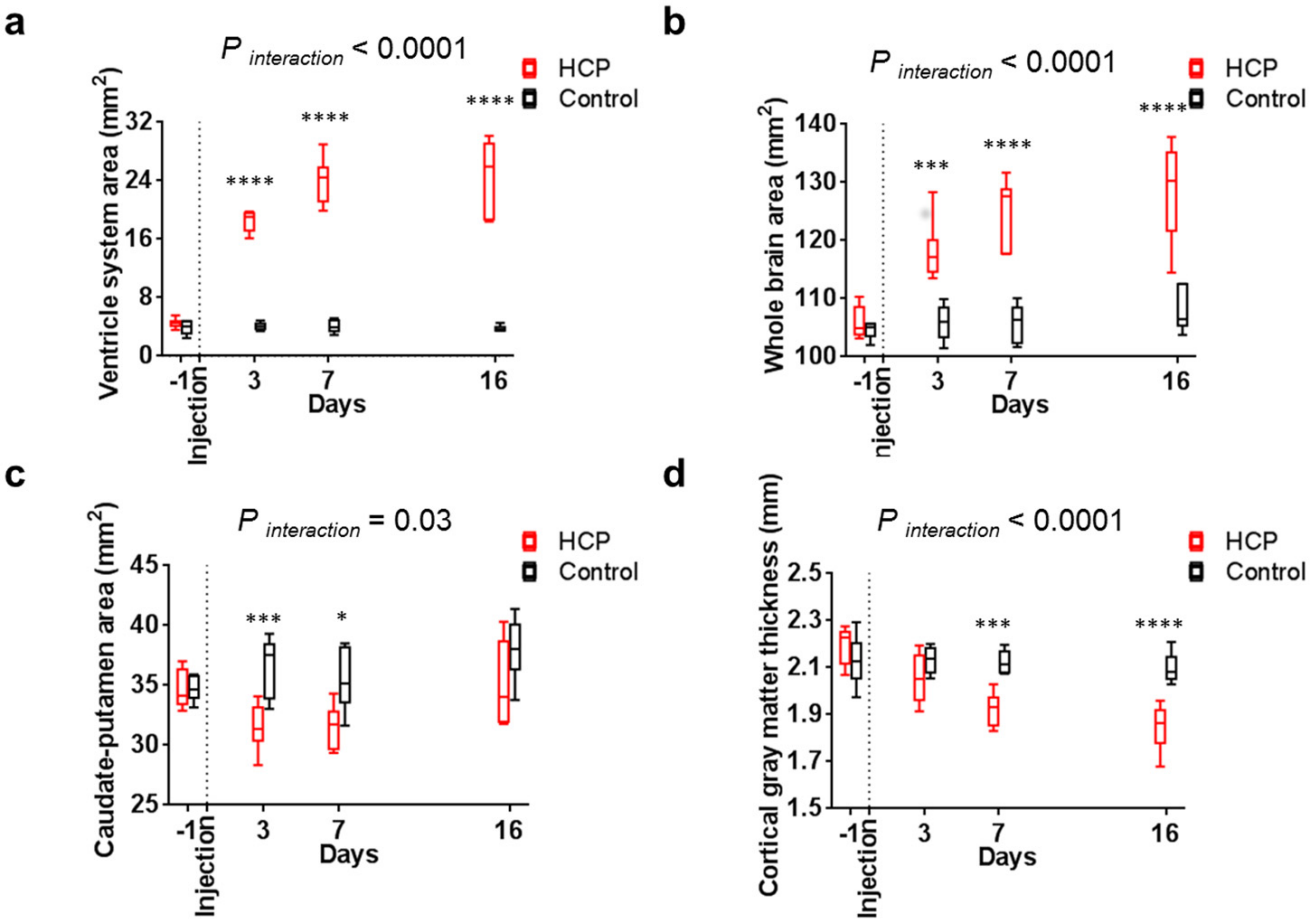
Brain deformation during hydrocephalus development. Brain deformation of hydrocephalic rats (□) were characterized from day three by a significantly (a) higher ventricle system cross-sectional area and (b) whole brain cross-sectional area, (c) a smaller caudate-putamen cross-sectional area at day three and day seven and also (d) a thinner cortical gray matter from day seven than control rats (□). *P* values for significance of the interaction between groups in the RM two-way ANOVA tests and significant Sidak’s comparisons are reported for each graph.

The cross-sectional area of the ventricular system and whole brain increased significantly over time in hydrocephalic rats, but not in controls (Fig 3, Table 1). This resulted in a significantly larger ventricular system and whole brain cross-sectional area at each time point post kaolin injection than in controls (Table 2).

**Table 1:** Summary of statistical evaluation of the effect of time on brain deformation in hydrocephalic and control rats.

	Brain deformation variables	Ventricular system cross-sectional area	Whole brain cross-sectional area	Caudate-putamen cross-sectional area	Cortical gray matter thickness
**Hydrocephalic rats (*P* value**^a^**)**	day -1 vs day 3	< 0.0001	< 0.0001	0.02	0.001
	day -1 vs day 7	< 0.0001	< 0.0001	0.05	< 0.0001
	day -1 vs day 16	< 0.0001	< 0.0001	0.94	< 0.0001
**Control rats (*P* value**^a^**)**	day -1 vs day 3	0.99	0.85	0.04	0.97
	day -1 vs day 7	0.99	0.86	0.33	0.90
	day -1 vs day 16	0.99	0.33	0.002	0.51

^a^ *P* values were calculated using Tukey’s post-hoc test.

**Table 2:** Summary of statistical evaluation of the effect of hydrocephalus induction on the brain deformation variables at each time point.

	Hydrocephalus vs control rats at each time-point(*P* value^a^)			
Brain deformation variables	Ventricular system cross-sectional area	Whole brain cross-sectional area	Caudate-putamen cross-sectional area	Cortical gray matter thickness
day -1	0.98	0.98	0.96	0.88
day 3	< 0.0001	0.0001	0.001	0.28
day 7	< 0.0001	< 0.0001	0.03	0.0004
day 16	< 0.0001	< 0.0001	0.12	< 0.0001

^a^ *P* values were calculated using Sidak’s post-hoc test.

The ventricles first enlarged rapidly, becoming 4.13 ± 0.39 times larger after the first three days. The enlargement then slowed, becoming 1.32 ± 0.19 times larger from day three to seven. The size of the ventricle reached equilibrium by day sixteen and did not enlarge any further. No significant difference in ventricular size was found between the last two time points (Tukey’s comparison, *P* = 0.88).

**Fig 4:**
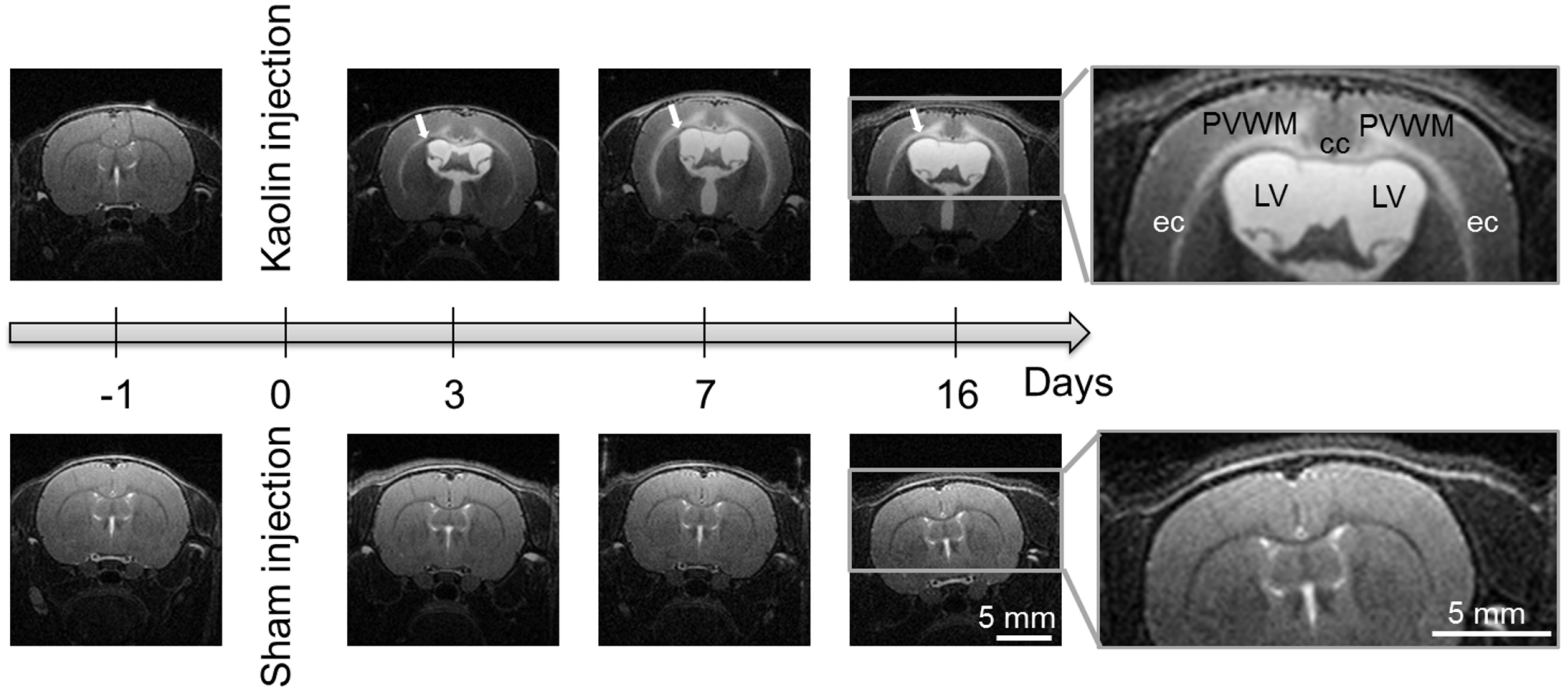
Typical T2-weighted anatomical MR images of rat brains obtained one day prior and three, seven, and sixteen days post kaolin injection (upper row) and sham injection (lower row). From day three, a hyperintense signal (indicated by a white arrow) covering the corpus callosum, periventricular white matter, the external capsule and the deep regions of the cortical gray matter was observed in hydrocephalic brains but not in controls, as seen on the magnified panels. This hyperintense signal was attributed to an increase in water content of the tissue. Note: The brightness of all images has been increased by 20% to improve the visibility for readers. Abbreviations: periventricular white matter (PVWM), corpus callosum (cc), external capsule (ec), and lateral ventricles (LV).

Ventricular enlargement was significantly associated with deformation of the caudate-putamen (GEE; *P* < 0.001) and cortical gray matter (GEE; *P* < 0.001). This was observed as a significant decrease in the cross-sectional area of the caudate-putamen at day 3, a progressive decrease of the thickness of the cortical gray matter, and an apparent flattening of the CC+PVWM (Fig 4) not seen in controls (Table 1). In the hydrocephalic rats, caudate-putamen cross-sectional area was lower at day three and day seven and cortical gray matter was also thinner than the controls from day seven (Fig 3).

### Diffusion properties

The fractional anisotropy provides information about the degree to which there is microstructural alignment (e.g. presence of aligned fiber tracts) in the tissue, while the mean diffusivity describes the overall ease of water diffusion, gives insight into the microstructural tissue features, and is increased in the present of edema.

#### Corpus callosum and periventricular white matter

**Fig 5:**
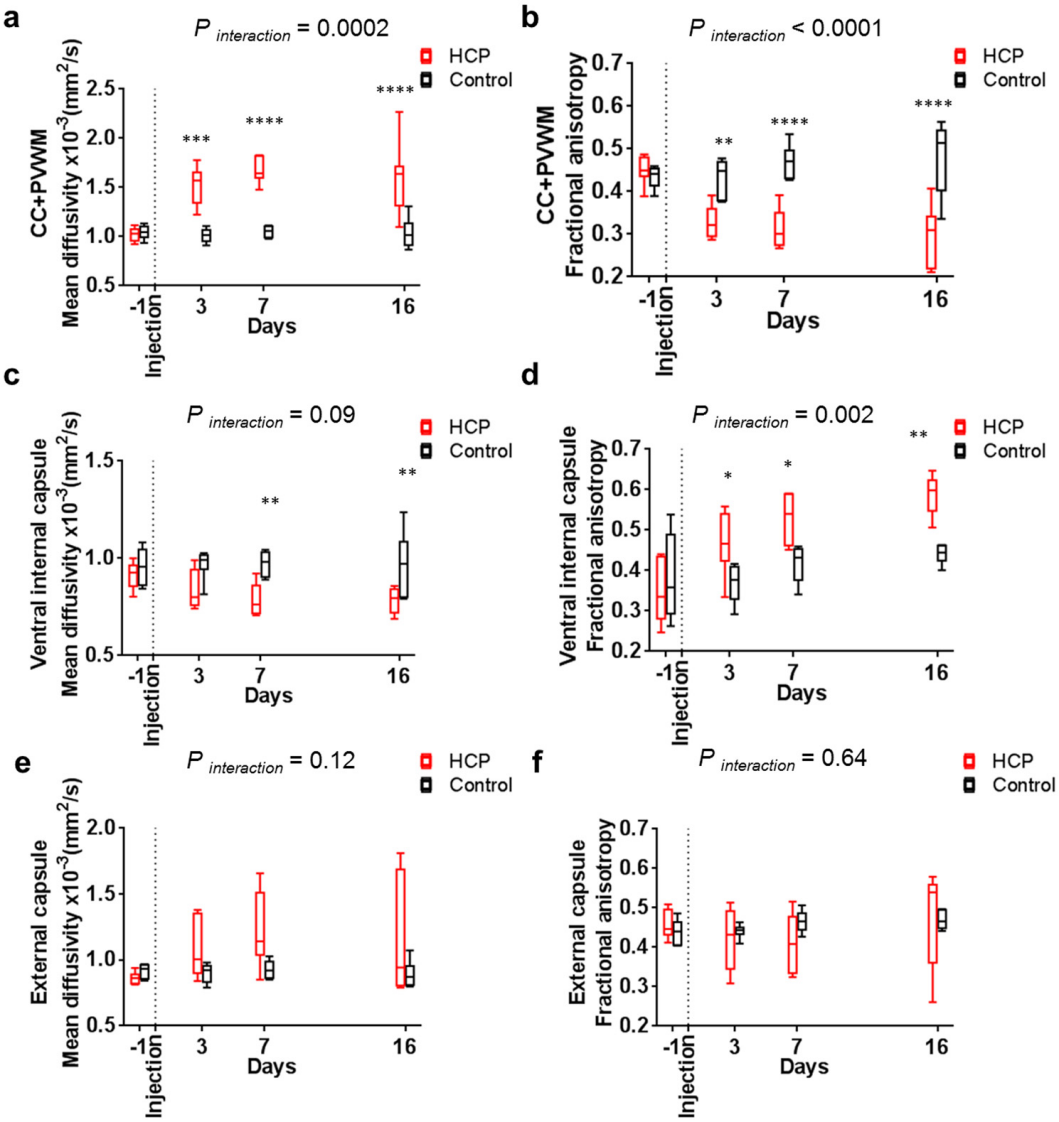
Changes in diffusion properties in the corpus callosum + periventricular white matter (CC+PVWM), ventral internal capsule and external capsule during hydrocephalus development. Diffusion properties in hydrocephalic rats (□) in the CC+PVWM (a) a higher mean diffusivity and (b) lower fractional anisotropy than for controls (□) from day three. In the ventral internal capsule, (c) the mean diffusivity was lower from day seven and (d) the fractional anisotropy was higher than for controls (□) from day three. *P* values for significance of the interaction between groups in the RM two-way ANOVA tests and significant Sidak’s comparisons are reported for each graph.

In the hydrocephalic rats, the mean diffusivity of CC+PVWM increased and the fractional anisotropy decreased significantly with ventricular enlargement (GEE, *P* < 0.001 for both), while there were no changes in controls (Fig 5, Tables 3 and 4). Mean diffusivity was higher and the fractional anisotropy was lower in the hydrocephalic rats than the controls from day three onwards (Tables 5 and 6).

**Table 3:** Summary of statistical evaluation of the effect of time on the mean diffusivity of brain tissue in hydrocephalic and control rats.

	Regions of interest	CC+PVWM^a^	Ventral internal capsule	External capsule	Cortical gray matter	Upper cortical gray matter	Caudate-putamen	Dorsal internal capsule
**Hydrocephalic rats (*P* value**^b^**)**	day -1 vs day 3	< 0.0001	0.50	0.11	0.67	0.83	0.46	0.60
	day -1 vs day 7	< 0.0001	0.01	0.04	0.002	0.94	0.29	0.99
	day -1 vs day 16	< 0.0001	0.009	0.03	0.001	0.93	0.53	0.62
**Control rats (*P* value**^b^**)**	day -1 vs day 3	0.99	0.91	0.99	0.94	0.67	0.87	0.87
	day -1 vs day 7	0.99	0.90	0.99	0.99	0.99	0.94	0.97
	day -1 vs day 16	0.99	0.93	0.99	0.91	0.63	0.73	0.82

^a^ CC+PVWM: corpus callosum and periventricular white matter.

^b^ *P* values were calculated using Tukey’s test.

**Table 4:** Summary of statistical evaluation of the effect of time on fractional anisotropy of the brain tissue in hydrocephalic and control rats.

	Regions of interest	CC+PVWM^a^	Ventral internal capsule	External capsule	Cortical gray matter	Upper cortical gray matter	Caudate-putamen	Dorsal internal capsule
**Hydrocephalic rats (*P* value**^b^**)**	day -1 vs day 3	0.0002	< 0.0001	0.70	0.03	0.01	0.56	< 0.0001
	day -1 vs day 7	< 0.0001	< 0.0001	0.73	0.0005	0.02	0.01	< 0.0001
	day -1 vs day 16	< 0.0001	< 0.0001	0.98	0.003	0.0004	< 0.0001	< 0.0001
**Control rats (*P* value**^b^**)**	day -1 vs day 3	0.99	0.98	0.99	0.57	0.95	0.47	0.99
	day -1 vs day 7	0.57	0.72	0.88	0.54	0.99	0.73	0.99
	day -1 vs day 16	0.33	0.30	0.85	0.94	0.99	0.22	0.99

^a^ CC+PVWM: corpus callosum and periventricular white matter.

^b^ *P* values were calculated using Tukey’s test.

**Table 5:** Summary of statistical evaluation of the effect of hydrocephalus induction on the mean diffusivity of brain tissue at each time point.

	Hydrocephalus vs control rats at each time-point(*P* value^b^)						
Regions of interest	CC+PVWM^a^	Ventral internal capsule	External capsule	Cortical gray matter	Upper cortical gray matter	Caudate-putamen	Dorsal internal capsule
day -1	0.99	0.99	0.99	0.82	0.60	0.50	0.63
day 3	0.0002	0.21	0.29	0.97	0.79	0.23	0.40
day 7	< 0.0001	0.002	0.19	0.13	0.92	0.01	0.28
day 16	< 0.0001	0.002	0.08	0.007	0.99	0.38	0.47

^a^ CC+PVWM: corpus callosum and periventricular white matter.

^b^ *P* values were calculated using Sidak’s post-hoc test.

**Table 6:** Summary of statistical evaluation of the effect of hydrocephalus induction on fractional anisotropy of brain tissue at each time point.

	Hydrocephalus vs control rats at each time-point(*P* value^b^)						
Regions of interest	CC+PVWM^a^	Ventral internal capsule	External capsule	Cortical gray matter	Upper cortical gray matter	Caudate-putamen	Dorsal internal capsule
day -1	0.97	0.09	0.99	0.81	0.99	0.52	0.99
day 3	0.004	0.02	0.93	0.10	0.009	0.68	0.0006
day 7	< 0.0001	0.008	0.55	0.002	0.04	0.006	< 0.0001
day 16	< 0.0001	0.0006	0.99	0.001	0.002	< 0.0001	< 0.0001

^a^ CC+PVWM: corpus callosum and periventricular white matter.

^b^ *P* values were calculated using Sidak’s post-hoc test.

#### Ventral internal capsule

In hydrocephalic rats, there was a significant decrease in mean diffusivity of the ventral internal capsule from day seven and an increase in the fractional anisotropy from day three as the ventricles enlarged (GEE, *P* < 0.001 for both), but there were no changes in controls (Fig 5, Tables 3 and 4). Mean diffusivity in the hydrocephalic rats was lower than the controls from day seven, and the fractional anisotropy was higher than the controls from day three (Tables 5 and 6).

#### External capsule

In hydrocephalic rats, mean diffusivity of the external capsule increased significantly from day seven as the ventricles enlarged (GEE, *P* < 0.001) but it did not change with time in controls (Fig 5, Table 2). Despite this increase in mean diffusivity observed in the hydrocephalic rats, the groups were not statistically different at all-time points in direct comparisons (Table 5). The fractional anisotropy of the external capsule in hydrocephalic rats did not change with ventricular enlargement, and was not significantly different than in controls at all time-points (Fig 5, Tables 4 and 6).

#### Cortical gray matter

**Fig 6:**
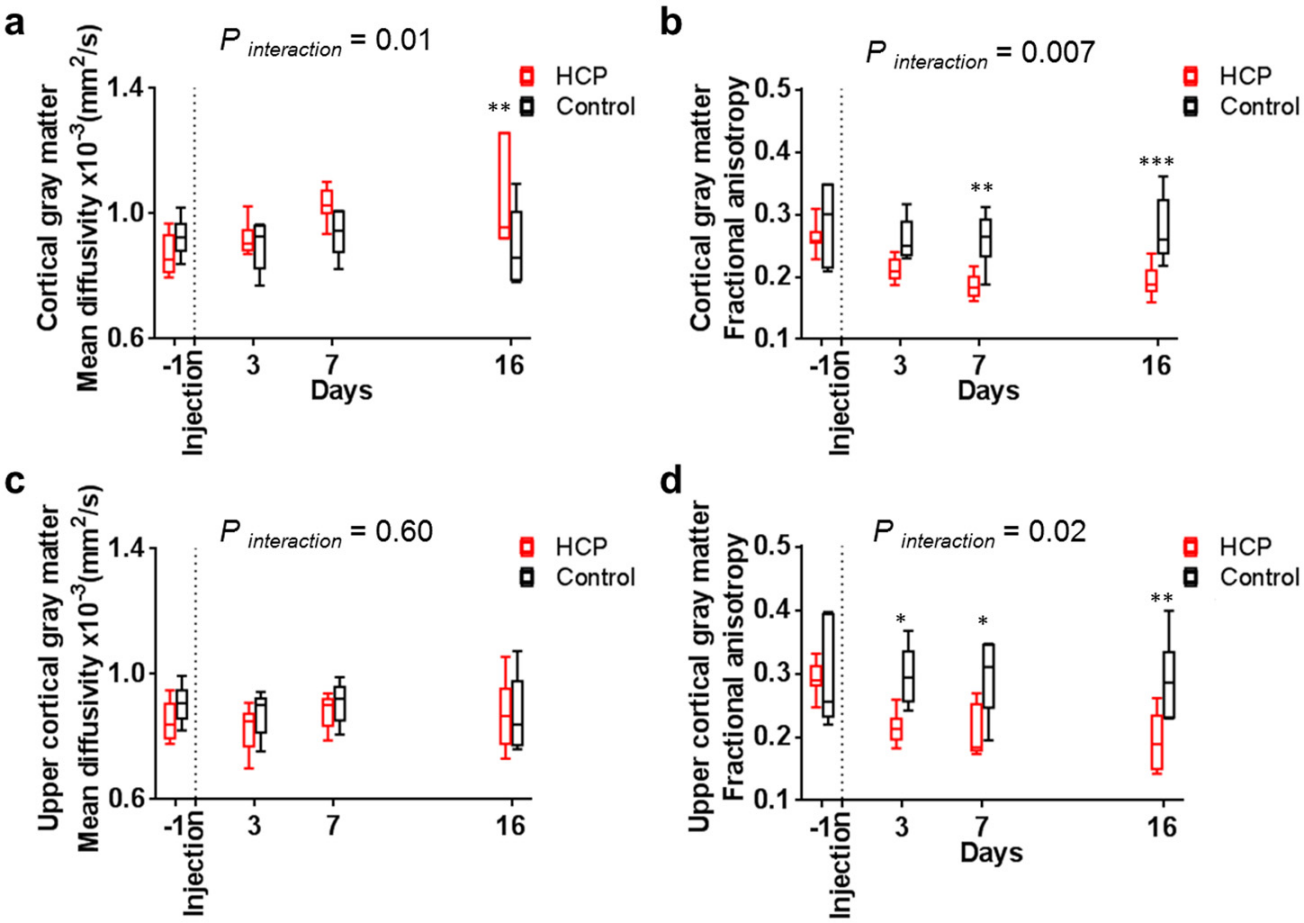
Changes in diffusion properties in cortical gray matter during hydrocephalus development. Diffusion properties of the cortical gray matter of hydrocephalic rats (□) had a (a) higher mean diffusivity than controls (□) at day sixteen and (b) lower fractional anisotropy than controls (□) from day seven. The upper cortical gray matter region in the presence of enlarged ventricles had (c) the same mean diffusivity as controls but a (d) lower fractional anisotropy from day three. *P* values for significance of the interaction between groups in the RM ANOVA tests and significant Sidak’s comparisons are reported for each graph.

The diffusion properties of the cortical gray matter in the hydrocephalic rats mirrored changes seen in the CC+PVWM. Mean diffusivity increased from day seven, and there was a decrease in fractional anisotropy after hydrocephalus induction (Fig 6, Tables 3 and 4). These changes were not seen in controls. In the hydrocephalic rats, mean diffusivity was higher than the controls at day sixteen and the fractional anisotropy was lower than the controls from day seven (Tables 5 and 6).

In contrast, the mean diffusivity in the upper cortical gray matter (i.e. superficial layers of the cortical gray matter, see Fig 2) did not change over time in either hydrocephalic or control animals and was not different between groups (Fig 6, Tables 3 and 5). However, the fractional anisotropy in the upper cortical gray matter decreased as the ventricles enlarged (GEE, *P* < 0.001) and was also significantly lower than the controls from day three (Fig 6, Tables 4 and 6).

#### Caudate-putamen

**Fig 7:**
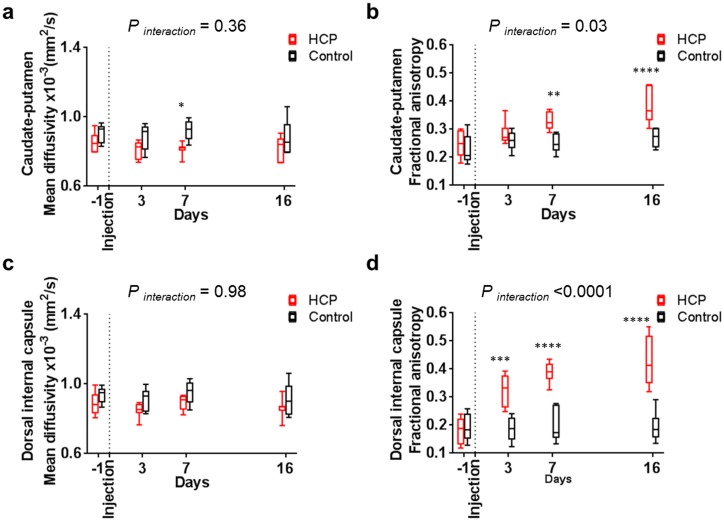
Changes in diffusion properties in the caudate-putamen during hydrocephalus development. Diffusion properties showed that the mean diffusivity of the (a) caudate-putamen and (c) dorsal internal capsule was similar in hydrocephalic rats (□) and controls (□). On the other hand, (b) the fractional anisotropy in caudate-putamen was significantly higher from day seven and (d) the dorsal internal capsule was significantly higher from day three in hydrocephalic rats (□) and controls (□). *P* values for significance of the interaction between groups in the RM ANOVA tests and significant Sidak’s comparisons are reported for each graph.

The mean diffusivity of the whole caudate-putamen and the dorsal internal capsule did not change with time in the hydrocephalic rats or the controls (Fig 7, Table 3). The fractional anisotropy increased significantly from day seven for the caudate-putamen and from day three for the dorsal internal capsule when the ventricle enlarged (GEE, *P* < 0.001 for both regions), while there were no changes in controls (Fig 7, Table 4). The fractional anisotropy of the caudate-putamen and dorsal internal capsule in the hydrocephalic rats was higher than the controls from day three (Table 6).

### Mechanical properties

#### Cortical gray matter

**Fig 8:**
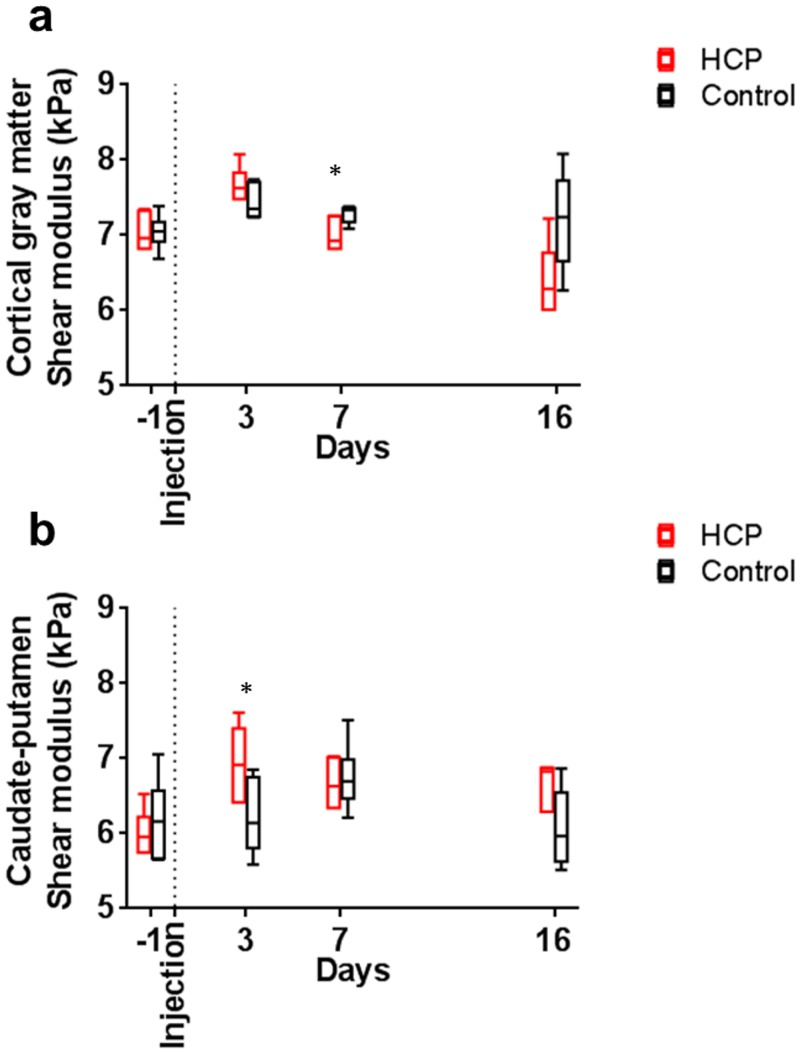
Changes in mechanical properties in the cortical gray matter and caudate-putamen during hydrocephalus development. Mechanical properties of the (a) cortical gray matter in hydrocephalic rats (□) showed a non-significant trend towards increased stiffness at day three and then decreased significantly by day seven, not seen in controls (□). In contrast, (b) the caudate-putamen stiffness in hydrocephalic rats was significantly higher at day three and then non-significant trended towards decreased stiffness. Significant t-tests are reported for each graph.

There was a non-significant trend towards increased stiffness at day three (t-test; day 3: *P* = 0.19) for the cortical gray matter. The stiffness then decreased significantly by day seven (t-test; day 7: *P* = 0.03, and day 16: *P* = 0.06, Fig 8). The decrease in shear modulus in the cortical gray matter was associated with the decrease in its thickness (GEE, *P* = 0.01), increased local mean diffusivity (GEE, *P* = 0.001), and increased cross-sectional area of the ventricular system (GEE, *P* = 0.02). However, the decrease in shear modulus was not associated with the decrease in the local fractional anisotropy (GEE, *P* = 0.10).

#### Caudate-putamen

In hydrocephalic rats, the stiffness of the caudate-putamen was significantly higher than the controls at day three (t-test; day 3: *P* = 0.03). There was then a non-significant trend towards decreased stiffness thereafter (t-test; day 7: *P* = 0.66, and day 16: *P* = 0.09, Fig 8). The increased shear modulus in the caudate-putamen was associated with a decreased cross-sectional area of the region (GEE, *P* = 0.05) and increased local fractional anisotropy (GEE, *P* = 0.001). However, no significant relationship was observed between stiffness of the caudate-putamen and the ventricle system cross-sectional area (GEE, *P* = 0.17).

### Histology

**Fig 9:**
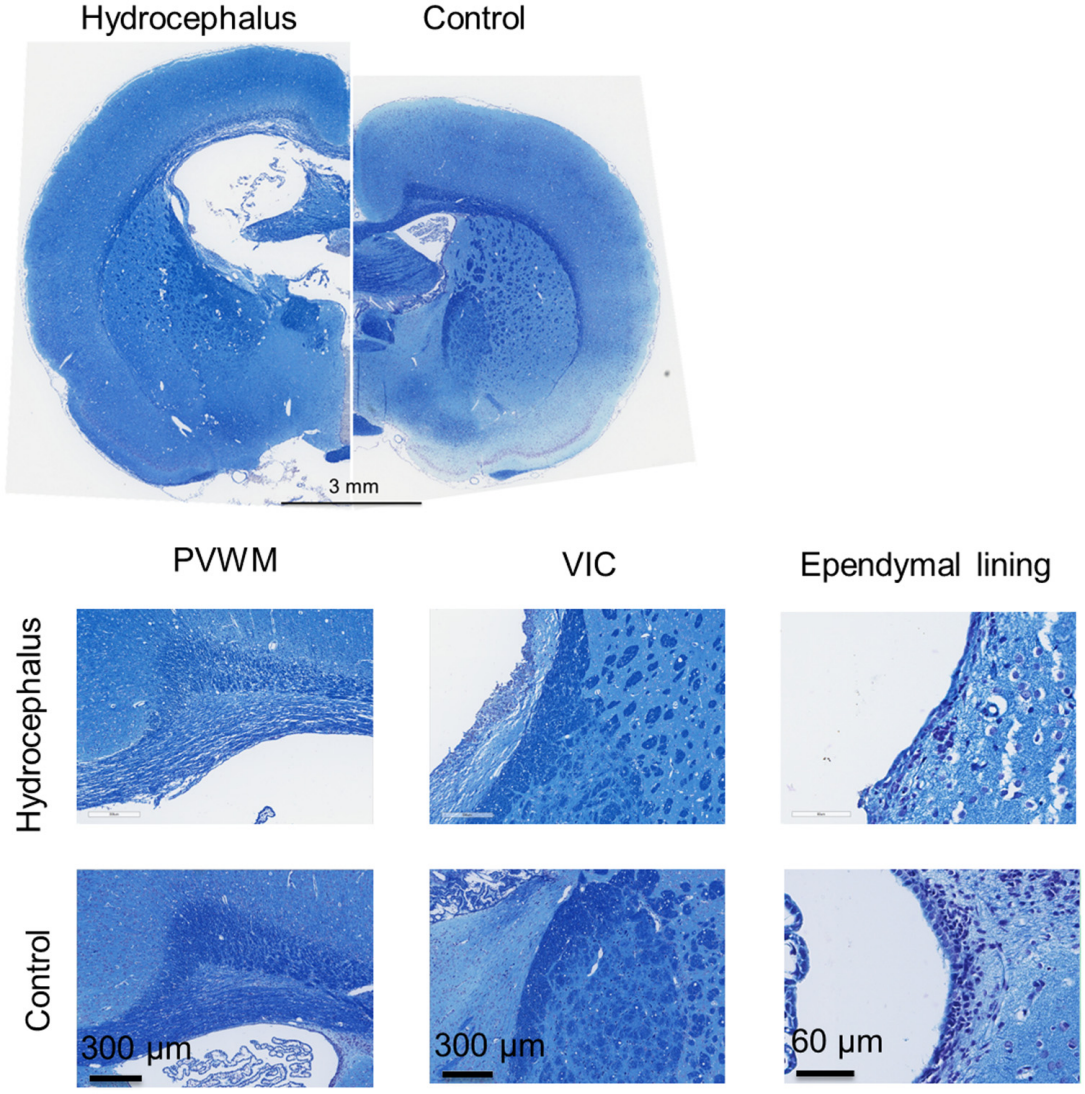
Typical histological sections stained with luxol fast blue and cresyl violet obtained from hydrocephalic and control brains sixteen days after kaolin / sham injection. In the hydrocephalic rats, macroscopic observations (top) showed a thinner cortical gray matter which seemed to have lost some of its ground surface in the area of the cingulum and a flattened caudate-putamen. In addition, on the higher magnitude panels (bottom), in hydrocephalic brain results showed the space between tracts increased in the corpus callosum and periventricular white matter. This was not seen in matching regions in controls. This was also not observed in the ventral internal capsule of hydrocephalic (c) and control rats. Finally, only in hydrocephalic rats, the ependymal cells lining the internal surface of the enlarged ventricles were flattened and their arrangement was discontinuous. Abbreviations: ventral internal capsule (VIC), periventricular white matter (PVWM).

Macroscopic observations of hydrocephalic rats’ brain at day sixteen showed enlargement of the ventricles and a degenerated septum pellucidum separated from the corpus callosum (Fig 9). In addition, the cortical gray matter was also thinner. Edema was demonstrated by the loss of ground surface in the area of the cingulum [47]. At the microscopic level, the ependymal cells lining the internal surface of the enlarged ventricles were flattened and their arrangement was discontinuous, especially along the roof of the lateral ventricles. Increased space between the connective tracts was observed in the CC+PVWM of hydrocephalic rats, but not in controls or in the ventral internal capsule of either hydrocephalic or control rats (Fig 9).

## Discussion

This study describes how the brain deforms during the development of hydrocephalus in a rat model. It also outlines the temporal changes in tissue microstructure and tissue stiffness in different brain regions as assessed by DTI and MR elastography, and how these changes are associated with ventricular enlargement.

### Ventricular enlargement and brain deformation

During the development of hydrocephalus, the cortical gray matter and the caudate-putamen deformed (Fig 3) significantly with ventricle size. In our rat model, the ventricles enlarged most rapidly between day one and day three, and reached equilibrium by day seven, with no significant further growth. The rapid ventricular enlargement in the initial stage of acute hydrocephalus development may be caused by the increase in CSF pressure due to the obstruction of CSF pathway, as suggested previously [47]. After this acute period, it has been previously shown, in a cat model, that transventricular absorption of CSF occurs [48, 49]. This may mitigate further increases in CSF pressure, and slow down ventricular enlargement. Our data suggest that a slowing in ventricular enlargement is not associated with an increase in brain stiffness (Fig 8), which rules out another possible explanation for the slowing, since a stiffer brain would require more pressure to further enlarge the ventricles.

The rapid increase in ventricle size during the initial stage of hydrocephalus development was compensated by the deformation of both the caudate-putamen and cortical gray matter. However, only the cortical gray matter continued to deform as the ventricular enlargement slowed. The reason for this is unclear and could be related to the proximity of the cortical gray matter to the calvarial sutures. The calvarial sutures in rats can remain patent throughout their life [50] and this allows intracranial volume to increase when the ventricles enlarge [51]. In this study, increased intracranial volume was demonstrated by the increase in whole brain cross sectional area (Fig 3) and the formation of a ‘dome’ on the top of the skull in hydrocephalic rats. As the caudate-putamen is adjacent to the ventricles but distant from the sutures, it has limited space to be displaced thus it tends to be compressed between the base of the skull and the enlarging ventricles during the initial stage of hydrocephalus. This is consistent with the observed increase in stiffness of the caudate-putamen during this period (Fig 8). At later time points, as the sutures allow intracranial volume to increase, and the gray matter displaces radially and thins, further compression of the caudate-putamen is minimal.

### Microstructural changes

Using DTI, changes in mean diffusivity can provide indirect information about microstructural features that alter the overall ease with which water molecules can diffuse. Modelling of the diffusion signal has been extensively used to quantify hindered and restricted diffusion in wide variety of tissue systems [52–54]. Measurements of diffusion anisotropy (e.g. fractional anisotropy) have also been used extensively to characterize neuronal organization in highly anisotropic white matter and less anisotropic gray matter. [23–25]. The diffusional anisotropy observed in white matter is generally attributed to the organized orientation of the white matter tracts, whereas radiating diffusion patterns that can be found in gray matter predominantly reflect ascending pyramidal cells and caudate-putamen to the nerve fibers of the striatum [25]. In hydrocephalus research, DTI has been used to characterize emergence of brain edema by a decrease in fractional anisotropy and increase in mean diffusivity [55, 56].

#### CC+PVWM and ventral internal capsule

In this study we observed a significant decrease in fractional anisotropy and concomitant increase in mean diffusivity in the CC+PVWM of hydrocephalic rats (Fig 5). This suggests that, due to brain edema, the extracellular space becomes larger and fiber structures might degenerate with progressing hydrocephalus, eventually leading to a reduction in diffusion restrictions in the space between the white matter tracts. These changes imply that the CC+PVWM microstructure was likely damaged during ventricular enlargement. These findings are consistent with the histology results and the presence of hyper-intense signal on the T2 weighted anatomical images (Fig 4), which has been attributed to the presence of edema [57]. Increase in water content of the CC+ PVWM may be caused by the infiltration of CSF from the ventricles after the destruction of the ependymal lining of the ventricles as previously suggested [58]. Although histology was only performed 16 days post-hydrocephalus induction in this study, previous studies suggest damage to the ependyma is likely to have commenced as early as the first 12 hours after hydrocephalus induction [59–61].

Changes in diffusion properties in the ventral internal capsule were different from those in the CC+PVWM even though both structures are close to the ventricles and in close proximity. We observed opposite diffusion behavior in the ventral internal capsule with an increase in fractional anisotropy and decrease in mean diffusivity (Fig 5), suggesting that in the ventral internal capsule, space between the white matter tracts was reduced. Unlike the CC+PVWM, which appeared to become degenerated, the ventral internal capsule was likely compressed probably at the expense of extracellular fluid. This opposite behavior in diffusion properties between the ventral internal capsule and the corpus callosum was consistent with observations in human hydrocephalic patients [62].

We observed regional variation in white matter microstructural changes not related to their proximity to the cerebral ventricles, similar to previous MR DTI studies [38, 63, 64]. While the CC+PVWM was damaged, the ventral internal capsule was compressed. Understanding this distinction is important because it is only possible to restore the function of the tissues using treatments such as shunts if the tissues are compressed and not degenerated or permanently damaged [65]. Previous studies have also shown specific recovery mechanisms in developing brains. For example, [64] showed that corticofugal pathways recovered quickly after intracranial pressure was lowered and this was likely related to the restoration of axoplasmic flow [64]. The development of indices based on changes in brain tissue diffusion properties are therefore useful as they are potential prognostic imaging indicators for the disease. Furthermore, our results showed that microstructural changes, indicative of neural degeneration or damage, in the CC+PVWM started within the first three days after hydrocephalus induction. It is therefore important to perform early treatment especially since damage to white matter is related to cognitive impairments in hydrocephalic patients, particularly in children [8, 66].

#### External capsule

The diffusion properties of the external capsule in our hydrocephalic rats did not change as much as the CC+PVWM and ventral internal capsule (Fig 5). This is probably because it is further away from the ventricles. Only a slight increase in mean diffusivity (Table 3) was observed. Together with the hyper-intense signal on the T2 weighted anatomical images, this suggests the presence of edema in this region. The unchanged longitudinal fractional anisotropy change indicates the absence of severe microstructural changes in the tissue. This finding was confirmed by our histological studies. One possible explanation for this could be that CSF in the ventricle leaked towards the external capsule after passing through the periventricular white matter.

The findings in this study are in contrast to observations previously made by two other diffusion imaging studies in hydrocephalic rats. One study described that the external capsule was compressed 4 weeks after kaolin injection in 3 weeks old rats [38]. Another study showed that the external capsule in rat pups injected with kaolin at post-natal day two was so severely damaged that it was almost unidentifiable on DTI maps [55]. The disparity in findings between those studies and our study is most likely related to the more severe ventriculomegaly in Yuan et al’s studies as larger ventricles are likely to impact neural structures further away from the ventricle. In our study, the ventricular system did not significantly increase in size between day 7 and 16, i.e. had reached a plateau by day 16 (Fig 3a and Table 1). This implies that even if we waited 4 weeks after hydrocephalus induction similar to the study of Yuan et al, these animals would probably not have ventriculomegaly large enough to alter the microstructure of the external capsule. On the other hand, brain tissue maturation in young rats also alters the tissue mechanical properties [67] and this may contribute to differences in the brain’s mechanical response to ventricular enlargement with age.

#### Cortical gray matter and caudate-putamen

The increase in mean diffusivity (Fig 6) and the presence of hyperintense signal on the T2 weighted anatomical images (Fig 4), together with the loss of ground substance in the area of the cingulum on the histological slices (Fig 9) suggest the presence of edema in the cortical gray matter. Similar to the CC+PVWM and the external capsule, this is likely due to an increase in extracellular tissue water from the ventricles via the discontinuous ependyma. The reasons for the decrease in fractional anisotropy in the cortical gray matter (Fig 6) could be due to an increase in extracellular space, alterations in tissue microstructural architecture or a combination of both. To better understand the specific changes in the cortical gray matter microstructure, diffusion properties were measured separately in an upper cortical gray matter region which excluded the edematous region adjacent to the corpus callosum and periventricular white matter. In this region, there was no significant change in the mean diffusivity over time but fractional anisotropy decreased significantly (Fig 6) and this suggests that extracellular space was not increased and changes were related to the alteration in tissue microstructural architecture. Similar observations have also been made in the cortical gray matter of H-Tx rats (a congenital model of hydrocephalus) where alteration of the tissue microstructural architecture was suggested to be related to the morphology of the dendritic structures such as decreases in dendritic length, number of dendritic branches and spine density [68].

Mean diffusivity of the caudate-putamen and dorsal internal capsule was not altered during ventricular enlargement and therefore, unlike the cortical gray matter, this brain region did not appear to be edematous (Fig 7). This was supported by the absence of hyperintense signal on the T2 weighted images (Fig 4). The increase in fractional anisotropy in these brain regions suggests a progressive compression of the caudate-putamen, beginning with the dorsal internal capsule, then extending to the entire caudate-putamen (Fig 7). Interestingly, the mean diffusivity in the caudate-putamen and dorsal internal capsule did not change despite it decreased in the ventral internal capsule. These findings suggest that, in contrast to the common belief [14, 69, 70], brain tissue compression does not occur everywhere in the hydrocephalus brain at the expense of extracellular fluid.

Changes in diffusion properties in the cortical gray matter and caudate-putamen were seen as early as three days post hydrocephalus induction and were similar than those reported on neonatal hydrocephalic rats [55]. This demonstrates that tissue microstructure was affected in the early stages of the disease. In addition, severity of the microstructural changes increased with ventricle size in both regions. The microstructural rearrangement is suspected to protect the cortical gray matter from injury when compressed [71, 72]. In fact, this cellular rearrangement may be important to allow geometry of the cortical gray matter to be restored after shunt treatment if the tissue is not degenerated [73]. Therefore, this study suggests that DTI may be a useful imaging technique to further investigate this unique mechanism of neural protection.

### Brain tissue mechanical properties

It is widely recognized that mechanical properties of neural tissues are related to their microstructure [74]. Hence, alterations in tissue microstructure may be reflected as an altered stiffness. Three days post hydrocephalus induction, the stiffness of the cortical gray matter and caudate-putamen increased (Fig 8) even though both regions underwent different changes in tissue microstructure. In the caudate-putamen, increased stiffness was associated with tissue compression and this was demonstrated by the increase in fractional anisotropy. Although cortical gray matter stiffness appeared to increase from day one to day three, this trend was not significant. In contrast, a previous study reported an increase in cortical gray matter stiffness in the same hydrocephalic rat model used in our study using an indentation technique [19]. There are two possible reasons for this disparity. First, brain stiffness measured using indentation through the dura includes dura stiffness which is much stiffer than the brain parenchyma and this may explain why stiffness reported in the study of Shulyakov was much higher (140–280 kPa) than our study (3–7 kPa). Second, measurement of brain stiffness may also be influenced by changes in CSF pressure in the extradural indentation experiments, while the elastography analysis method used here removes the contribution of hydrostatic pressure on mechanical properties, quantifying the shear modulus [42].

In contrast, when the ventricles enlarged more slowly in the later stages of hydrocephalus development, regional variations in mechanical properties seem to reflect the alteration of the tissue microstructure and water content. For example, in the cortical gray matter, the stiffness was lower in hydrocephalic brains than in controls (Fig 8). In the deeper cortical grey matter overlying the ventricles, the decrease in stiffness was associated with edema (Fig 6). However, in the more superficial grey matter region, where edema was not significant, no correlation between stiffness and decrease in fractional anisotropy (Fig 6) was found. A possible interpretation for this finding is that microstructural rearrangement occurs in these regions and this suggests that the increase in extracellular water may have softened the tissue. This is consistent with a recent study which demonstrates that the presence of CSF in neural tissue decreases the stiffness as measured by MR elastography [75]. Softening of the brain tissue in the presence of enlarged ventricles has previously been observed in normal pressure hydrocephalus, however the authors suggested that this change is related to tissue degeneration [16]. Therefore, it is likely that different microstructural alterations can have different effects on tissue stiffness.

Finally, the magnitude of the stiffness changes reported in hydrocephalic rats (~1kPa, or ~15% increase from a baseline of 6kPa) seemed relatively modest. However, the only study to have quantified the effect of tissue compression on measured stiffness using MR elastography showed that 10–15% of uniform compression (similar in magnitude to that is seen in the cortical grey matter here) in ex vivo liver tissue increased the apparent stiffness by ~30–40% [76]. This was also reported in another study using shear rheometry [77]. Given that the brain regions were not uniformly compressed throughout the ROIs used in this study, average increases in stiffness of the order of 10–15% are broadly consistent with what might be expected from the known nonlinear rheological properties of brain tissue [74].

The changes in mechanical properties and diffusion properties observed during ventricular enlargement highlight the complementary nature of DTI and MR elastography as imaging techniques for assessing brain tissue microstructural change. This is particularly obvious in the caudate-putamen where the shear modulus was significantly increased on day 3 but not on day 7 and 16 (Fig 8b), while the fractional anisotropy significantly decreased only from day 7 (Fig 7b). This difference in time-course maybe because the two imaging techniques are not sensitive to the “same” microstructural alterations. For DTI, it is well accepted that cytopathological changes that affect the neuronal microstructure differently in different directions are usually reflected by changes in FA. An example in the current study is in the caudate-putamen, where the progressive increase in fractional anisotropy with time most likely reflects tissue compression. However, for MR elastography, the relationship between tissue stiffness and tissue microstructure is not yet well understood, and few studies linking mechanical property changes in different brain regions to underlying tissue microstructure changes have been performed. In this study, we measured an increase in caudate-putamen stiffness when the ventricles enlarged rapidly, but this was not seen when the ventricles enlarged more slowly. The reasons for this are not fully clear from our study, but it may be that there is more time for tissue relaxation during the slower loading than during the more rapid loading. This could occur due to microstructural reorganization that does not affect the diffusion fractional anisotropy, fluid redistribution, or inherent neural tissue viscoelastic behaviour. Further studies are required to determine the mechanisms of these differences.

### Limitations

There are some limitations that are inherent to the imaging techniques used in this study. Due to the anatomy of the skull-CSF-brain interface it is generally difficult to achieve high wave amplitudes for MR elastography inside the brain. The attenuation of mechanical wave through the skull and CSF is a common problem in brain MR elastography. In our study, this resulted in exclusion of ten MR elastography data sets due to low wave amplitude in the brain. As a result, we used paired t-tests to compare groups at each time point. This approach does not consider the effect of repeated measurements, and does not allow for analysis of changes in brain mechanical properties over time. Nevertheless, we were able to obtain consistent data in the remaining datasets which are in line with brain mechanical properties measured using similar techniques in the literature [31–35].

Furthermore, as hydrocephalus has been widely considered to be a disease affecting the periventricular white matter, it would be interesting to study how brain tissue mechanical properties change in the white matter. However, it was difficult to measure the shear modulus of these small brain regions in the rat brain due to spatial resolution limits. Although spatial resolution can be increased, it would require substantially longer acquisition times, which are undesirable for the well-being of anaesthetized animals. In addition, MR imaging was only performed at the foramina of Monro, which did not allow analyses of other brain regions which could contribute to the regional differences observed since several of the important white matter tracts are located in the more caudal parts of the brain.

It would also be interesting to quantify the deformation of the CC + PVWM, internal and external capsule over time. Unfortunately, it was not possible to accurately segment these regions on the T2 weighted anatomical images as the white matter regions were poorly distinguishable either due to the emergence of edema covering the superficial brain regions in hydrocephalic rats, or due to the lack of contrast for the ventral internal capsule. Further improvements in imaging hardware and protocols might facilitate such measurements. Volumetric measurements could also contribute to the discussion about the regional tissue differences observed. However, these measurements could not be done on the anatomical scans as only 9 slices of 300 μm of thickness were acquired. It was also not possible to add a dedicated acquisition due to limited scan time (~ 2 hours per rat). Extending the scan time of our protocol would have been detrimental to the well-being of the animals.

Further histological and immunohistological staining could also be performed to determine the extent of astrocytosis and microgliosis to further understand how these may affect tissue stiffness [78], as changes in diffusion properties correlated with cytopathological changes in a rat model of hydrocephalus [55]. However, this was not investigated in the current work but would be interesting to investigate in future studies. In this study, Luxol Fast Blue and Cresyl violet staining were performed primarily to show that our experimental hydrocephalus model was similar to existing studies, in which more detailed histological analyses have been performed.

Finally, we did not measure intracranial pressure, blood pressure or arterial blood gases in this study even though they areimportant factors to consider in thedevelopment of obstructive hydrocephalus. Intracranial pressure measurements involve opening the cranial vault which would likely alter the disease development, and are difficult to combine with the MR elastography technique without placing undue stress on the animals. Further experiments are needed to determine if changes in intracranial pressure, blood pressure and arterial blood gases during hydrocephalus development are related to changes in brain stiffness, particularly during the rapid initial ventricular enlargement.

## Conclusions

This study showed that MR elastography and DTI are able to track brain microstructure and mechanical property changes during the development of obstructive hydrocephalus. This is the first time that brain mechanical properties have been measured in vivo and non-invasively in such conditions and results demonstrated that changes in stiffness were different in the presence of edematous or compressed tissue and rapid or slow ventricular enlargement. The mechanical and diffusion property changes followed distinct time courses during the development of hydrocephalus, suggesting that they have the potential to be complementary imaging biomarkers for tracking the effects of brain deformation on the underlying brain tissue microstructure which is not visible on conventional anatomical MR images. However, whilst diffusion changes were larger and statistically significant for majority of the brain regions studied, the changes in mechanical properties were more modest, and occurred primarily in the acute phase of hydrocephalus development.

In conclusion, by using these imaging methods, we investigated the progression of hydrocephalus and showed that in the rat model used in this study (1) the impact of ventricular enlargement is not limited to the corpus callosum and periventricular white matter in the early stages of hydrocephalus development, (2) the extent of microstructural changes depends on the brain region and varies during hydrocephalus development, and (3) there is a regional and temporal variation in brain tissue stiffness during hydrocephalus development.

## Supporting Information

S1 Table Mean and standard deviation of the brain deformation variables obtained in hydrocephalic and controls rats. (PDF)

S2 Table Mean and standard deviation of the brain mean diffusivity obtained in hydrocephalic and controls rats. (PDF)

S3 Table Mean and standard deviation of the brain fractional anisotropy obtained in hydrocephalic and controls rats. (PDF)

S4 Table Mean and standard deviation of the brain shear modulus in hydrocephalic and controls rats. (PDF)
