# Supplementary material for: Changes in Rat Brain Tissue Microstructure and Stiffness during the Development of Experimental Obstructive Hydrocephalus
Source: PLoS One. 2016 Feb 5;11(2):e0148652. doi: 10.1371/journal.pone.0148652 (PMC4743852; doi:10.1371/journal.pone.0148652)
Supplement: S1 Table — (PDF) [file pone.0148652.s001.pdf]

S1 Table. Mean and standard deviation of the brain deformation variables obtained in hydrocephalic and controls rats.

Brain deformation variables		Baseline	Post-hydrocephalus Induction		
		Day -1	Day 3	Day 7	Day 16
**Ventricular system cross-sectional area (mm^2)**	Controls	3.8 ± 0.9	4.1 ± 0.5	4.0 ± 0.9	3.8 ± 0.4
	Hydrocephalus	4.5 ± 0.6	18.5 ± 1.3	24.2 ± 3.1	25.0 ± 4.8
**Whole brain cross-sectional area (mm^2)**	Controls	105 ± 1	106 ± 3	106 ± 3	108 ± 4
	Hydrocephalus	106 ± 3	118 ± 5	125 ± 6	128 ± 8
**Caudate-putamen cross-sectional area (mm^2)**	Controls	35 ± 1	37 ± 2	35 ± 3	38 ± 3
	Hydrocephalus	35 ± 2	31 ± 2	32 ± 2	35 ± 3
**Cortical gray matter thickness (mm)**	Controls	2.13 ± 0.11	2.13 ± 0.06	2.12 ± 0.05	2.10 ± 0.06
	Hydrocephalus	2.20 ± 0.08	2.05 ± 0.10	1.92 ± 0.07	1.85 ± 0.09
