# Supplementary material for: Changes in Rat Brain Tissue Microstructure and Stiffness during the Development of Experimental Obstructive Hydrocephalus
Source: PLoS One. 2016 Feb 5;11(2):e0148652. doi: 10.1371/journal.pone.0148652 (PMC4743852; doi:10.1371/journal.pone.0148652)
Supplement: S2 Table — (PDF) [file pone.0148652.s002.pdf]

S2 Table. Mean and standard deviation of the brain mean diffusivity obtained in hydrocephalic and controls rats.

Mean diffusivity × 10^{-3} mm^2/s (mean ± standard deviation)		Baseline Day -1	Post-hydrocephalus Induction Day 3	Day 7	Day 16
**Corpus callosum + Periventricular white matter**	Controls	1.05 ± 0.07	1.01 ± 0.07	1.05 ± 0.06	1.04 ± 0.15
	Hydrocephalus	1.02 ± 0.07	1.52 ± 0.19	1.67 ± 0.12	1.58 ± 0.38
**Ventral internal capsule**	Controls	0.96 ± 0.09	0.97 ± 0.08	0.97 ± 0.06	0.97 ± 0.17
	Hydrocephalus	0.91 ± 0.07	0.83 ± 0.10	0.78 ± 0.08	0.78 ± 0.06
**External capsule**	Controls	0.92 ± 0.05	0.90 ± 0.07	0.93 ± 0.07	0.89 ± 0.10
	Hydrocephalus	0.86 ± 0.04	1.09 ± 0.22	1.22 ± 0.28	1.17 ± 0.43
**Cortical gray matter**	Controls	0.93 ± 0.06	0.90 ± 0.08	0.94 ± 0.08	0.89 ± 0.12
	Hydrocephalus	0.87 ± 0.06	0.92 ± 0.05	1.05 ± 0.05	1.06 ± 0.17
**Upper Cortical gray matter**	Controls	0.92 ± 0.07	0.87 ± 0.07	0.91 ± 0.07	0.87 ± 0.12
	Hydrocephalus	0.85 ± 0.06	0.82 ± 0.07	0.88 ± 0.06	0.88 ± 0.11
**Caudate-putamen**	Controls	0.91 ± 0.05	0.89 ± 0.07	0.92 ± 0.06	0.88 ± 0.10
	Hydrocephalus	0.85 ± 0.05	0.81 ± 0.05	0.81 ± 0.04	0.82 ± 0.07
**Dorsal internal capsule**	Controls	0.94 ± 0.04	0.91 ± 0.06	0.95 ± 0.07	0.91 ± 0.10
	Hydrocephalus	0.89 ± 0.06	0.85 ± 0.04	0.89 ± 0.04	0.86 ± 0.06
