# Supplementary material for: Changes in Rat Brain Tissue Microstructure and Stiffness during the Development of Experimental Obstructive Hydrocephalus
Source: PLoS One. 2016 Feb 5;11(2):e0148652. doi: 10.1371/journal.pone.0148652 (PMC4743852; doi:10.1371/journal.pone.0148652)
Supplement: S3 Table — (PDF) [file pone.0148652.s003.pdf]

S3 Table. Mean and standard deviation of the brain fractional anisotropy obtained in hydrocephalic and controls rats.

Fractional anisotropy (mean ± standard deviation)		Baseline	Post-hydrocephalus Induction		
		Day -1	Day 3	Day 7	Day 16
**Corpus callosum + Periventricular white matter**	Controls	0.43 ± 0.03	0.43 ± 0.04	0.47 ± 0.04	0.48 ± 0.09
	Hydrocephalus	0.45 ± 0.03	0.33 ± 0.04	0.31 ± 0.05	0.29 ± 0.07
**Ventral internal capsule**	Controls	0.38 ± 0.11	0.37 ± 0.05	0.42 ± 0.05	0.44 ± 0.02
	Hydrocephalus	0.35 ± 0.08	0.47 ± 0.07	0.53 ± 0.06	0.59 ± 0.05
**External capsule**	Controls	0.44 ± 0.03	0.44 ± 0.02	0.47 ± 0.03	0.47 ± 0.02
	Hydrocephalus	0.46 ± 0.04	0.42 ± 0.08	0.42 ± 0.07	0.47 ± 0.12
**Cortical gray matter**	Controls	0.27 ± 0.06	0.26 ± 0.03	0.26 ± 0.04	0.28 ± 0.05
	Hydrocephalus	0.26 ± 0.02	0.21 ± 0.02	0.18 ± 0.02	0.20 ± 0.03
**Upper Cortical gray matter**	Controls	0.29 ± 0.08	0.30 ± 0.05	0.30 ± 0.06	0.29 ± 0.06
	Hydrocephalus	0.29 ± 0.03	0.22 ± 0.02	0.21 ± 0.04	0.19 ± 0.04
**Caudate-putamen**	Controls	0.23 ± 0.05	0.26 ± 0.03	0.25 ± 0.03	0.27 ± 0.03
	Hydrocephalus	0.25 ± 0.05	0.28 ± 0.04	0.33 ± 0.03	0.38 ± 0.06
**Dorsal internal capsule**	Controls	0.19 ± 0.05	0.19 ± 0.04	0.20 ± 0.06	0.19 ± 0.05
	Hydrocephalus	0.18 ± 0.05	0.32 ± 0.05	0.39 ± 0.04	0.43 ± 0.09
