# Supplementary material for: Changes in Rat Brain Tissue Microstructure and Stiffness during the Development of Experimental Obstructive Hydrocephalus
Source: PLoS One. 2016 Feb 5;11(2):e0148652. doi: 10.1371/journal.pone.0148652 (PMC4743852; doi:10.1371/journal.pone.0148652)
Supplement: S4 Table — (PDF) [file pone.0148652.s004.pdf]

S4 Table. Mean and standard deviation of the brain shear modulus in hydrocephalic and controls rats.

Shear modulus (kPa) (mean ± standard deviation)		Baseline	Post-hydrocephalus Induction		
		Day -1	Day 3	Day 7	Day 16
**Cortical gray matter**	Controls	7.0 ± 0.2	7.4 ± 0.2	7.3 ± 0.1	7.2 ± 0.7
	Hydrocephalus	7.0 ± 0.2	7.7 ± 0.2	7.0 ± 0.2	6.4 ± 0.5
**Caudate-putamen**	Controls	6.2 ± 0.5	6.2 ± 0.5	6.7 ± 0.4	6.1 ± 0.5
	Hydrocephalus	6.0 ± 0.3	6.9 ± 0.5	6.6 ± 0.4	6.7 ± 0.4
